# Nutrition Support in Dysphagia: Japan Nationwide Hospital Survey on Nutritional Values, Diet Characteristics, and Dietitians' Roles in Texture-Modified Diets

**DOI:** 10.7759/cureus.86191

**Published:** 2025-06-17

**Authors:** Yuka Shirai, Junko Ueshima, Keisuke Maeda, Fumie Egashira, Yuri Horikoshi, Satoru Kamoshita, Ryo Momosaki

**Affiliations:** 1 Department of Rehabilitation Medicine, Mie University Graduate School of Medicine, Tsu, JPN; 2 Clinical Nutrition Unit, Hamamatsu University Hospital, Hamamatsu, JPN; 3 Japanese Working Group on Integrated Nutrition for Dysphagic People (JWIND), Tokyo, JPN; 4 Department of Nutritional Service, NTT Medical Center Tokyo, Tokyo, JPN; 5 Department of Geriatric Medicine, Hospital, National Center for Geriatrics and Gerontology, Obu, JPN; 6 Nutrition Therapy Support Center, Aichi Medical University, Nagakute, JPN; 7 Home Nutrition Services, PEACH Atsugi, Community Nutritional Care Center, Atsugi, JPN; 8 Medical Affairs Department, Research and Development Center, Otsuka Pharmaceutical Factory, Inc., Tokyo, JPN

**Keywords:** dietitian, dysphagia, nutrient, nutrition support, texture-modified diets

## Abstract

This study aimed to investigate the current nutrition support practices for patients with dysphagia in Japanese hospitals and to identify gaps between research-based recommendations and clinical practice. A nationwide questionnaire survey was conducted from April to June 2023 targeting 5,376 hospitals with inpatient beds. The questionnaire, developed by the Japanese Working Group on Integrated Nutrition for Dysphagic People, included items on hospital characteristics and nutrition support systems. Hospitals that consented to participate completed an online questionnaire by a representative dietitian. A total of 905 hospitals responded (response rate: 16.8%). Of these, 94.7% provided texture-modified diets (TMDs). Among 857 hospitals providing TMDs, 511 (59.6%) reported that nutrition support for patients with dysphagia was provided by ward-assigned dietitians. In contrast, 229 hospitals (26.7%) had no clearly designated personnel responsible for such support. Multidisciplinary teams were absent in 58.7% of hospitals. While 84.8% of hospitals reported discrepancies between the names and actual forms of TMDs, only 37.2% shared dietary forms with other facilities. The median energy content of TMDs ranged from 1,200 to 1,400 kcal/day, suggesting inadequate nutritional value. This study revealed insufficient energy content in TMDs and limited use of standardized dietary forms, despite recognition of these issues by nutrition departments. The lack of multidisciplinary collaboration and inconsistent classification may hinder effective nutrition support. Addressing organizational, structural, and communication-related gaps is essential to improve the quality of dysphagia nutrition care.

## Introduction

Nutrition support plays a critical role in the clinical management of patients with dysphagia. The prevalence of undernutrition in this population has been reported to reach 45.3%, regardless of patients’ functional status or comorbidities [1]. Undernutrition is associated with prolonged hospitalization, increased risk of infection, delayed wound healing, higher healthcare costs, and poorer clinical outcomes [2,3]. Therefore, appropriate nutrition support is essential for patients with dysphagia. Such support includes liquid thickening, modification of diet texture, food fortification, and the use of oral nutritional supplements [4]. These interventions may improve swallowing safety and efficiency while helping to prevent nutritional complications such as inadequate nutrient intake and weight loss [5,6]. When properly implemented, these strategies may contribute to improved treatment outcomes.

Texture-modified diets (TMDs) are a cornerstone of nutrition support for patients with dysphagia. These diets are specifically designed to ensure safe and effective eating and swallowing [5] and are also expected to support improved nutritional status. In patients with dysphagia, functional impairments often limit food consumption. In addition, TMDs are prone to nutrient loss due to added water and processing during preparation [7]. As such, ensuring sufficient energy intake is critical for preventing undernutrition [4]. To address these concerns, recent studies have demonstrated the effectiveness of nutrient-dense TMDs. These fortified diets have been shown to enhance nutrient intake without significantly affecting hunger, satiety, or appetite, and have also been reported to improve nutritional status and physical function [8,9]. Therefore, TMDs that consider both energy content and nutrient density are essential for delivering high-quality nutrition support to patients with dysphagia.

However, nationwide data on the implementation of TMDs and related nutritional practices in Japan remain scarce. Although classification systems developed by academic societies are being used to standardize food textures, these frameworks primarily focus on physical properties, with limited reference to nutritional criteria such as energy and nutrient content [10]. Consequently, even when facilities comply with these classifications, the nutritional value of TMDs may vary. Moreover, it is unclear how consistently these classification-based standards are applied in actual clinical settings. Clarifying this discrepancy between classification systems and real-world practice, the so-called evidence-practice gap, is essential for improving and standardizing nutrition support for patients with dysphagia. Furthermore, the prevalence of dysphagia increases with age [11], and Japan, as one of the world’s most rapidly aging societies, has the highest aging rate in Asia. Understanding the current situation in Japan and disseminating the findings internationally may provide a valuable foundation for developing nutrition policies in other countries facing similar demographic transitions.

This study aims to investigate the current nutrition support practices for patients with dysphagia in Japanese hospitals and to identify gaps between research-based recommendations and actual clinical practice.

## Materials and methods

Study population

This study targeted the nutrition departments of 5,376 hospitals across Japan that had inpatient beds, regardless of hospital size or geographic location. These hospitals were registered as acute care hospitals in the National Registry of Medical Institutions. In April 2023, a survey request regarding nutrition support for patients with dysphagia was distributed to dietitians working at these hospitals. Respondents were informed that online registration would be considered as providing consent to participate in the study. The survey employed a self-selection sampling method, in which only hospitals that voluntarily agreed to participate submitted responses. This study was approved by the Ethics and Conflict of Interest Committee of the National Center for Geriatrics and Gerontology (Approval No. 1673).

The Japan Working Group for Integrative Nutrition of Dysphagic Patients

We established the Japan Working Group for Integrative Nutrition of Dysphagic Patients (JWIND), an independent expert group, in December 2021 to generate evidence for nutrition support in eating and swallowing and to develop new nutrition support strategies for adult patients with dysphagia. JWIND comprises physicians (geriatric and rehabilitation medicine) and dietitians, mainly Certified Specialist of Registered Dietitian for Dysphagia Rehabilitation [12], a joint certification system of the Japan Dietetic Association and the Japanese Society of Dysphagia Rehabilitation [4].

Survey strategy 

The questionnaire used in this study was developed by JWIND and was specifically designed for this survey. It consisted of a total of 25 questions covering hospital characteristics, staffing, dysphagia management practices, and the nutritional content of TMDs (Appendix A). Five researchers with expertise in clinical nutrition and dysphagia evaluated the clarity, validity, and structure of the questionnaire items. Through repeated discussions, they thoroughly reviewed and revised the content, resulting in the finalized version of the questionnaire. However, formal reliability testing, such as test-retest reliability and internal consistency analysis, was not conducted. The questionnaire included a combination of question formats: multiple-choice questions (e.g., yes/no, single- and multiple-answer), numerical entry fields (e.g., number of hospital beds, energy and protein content), and open-ended questions for additional comments or clarifications. This design enabled respondents to provide both standardized and facility-specific information. The online system included input validation features and allowed respondents to review and revise their answers before submission. The survey was conducted online from April to June 2023. Request letters were mailed to the target hospitals, outlining the purpose of the survey on nutrition support for patients with dysphagia and providing a link to the online questionnaire. Hospitals that consented to participate completed an online registration process, and one representative from each hospital’s nutrition department responded to the survey. To improve the response rate, reminder emails and letters were sent to non-responding hospitals before the initial deadline. The survey deadline was also extended to ensure sufficient time for participation. The development, implementation, and data aggregation of the web-based questionnaire system were entrusted to an independent third-party organization (Macromill Carenet, Inc., Tokyo, Japan), separate from the study authors. All procedures were carried out in accordance with the Act on the Protection of Personal Information in Japan.

Study variables

Data collected in this study included hospital characteristics (type, number of beds, average length of stay, and number of dietitians); nutrition support systems for patients with dysphagia (assigned dietitians, presence of multidisciplinary teams, nutrition screening, and assessment methods); and nutritional characteristics of TMDs, including energy and protein content, recognition of nutritional value, provision criteria, and naming conventions. Responses were obtained using structured question formats. Dietitians were asked to enter actual numerical values for energy and protein content (kcal/day, g/day) based on the meals provided at their respective hospitals. No reference values were presented during the survey in order to objectively capture the nutritional content provided in actual clinical practice. However, for the purpose of analysis and evaluation, the Dietary Reference Intakes for Japanese (2020 edition) were used [13]. In particular, the estimated energy requirements for individuals aged 75 years and older were applied to assess the adequacy of the reported energy content. In this study, the Japanese Dysphagia Diet 2021 (JDD2021) was used to classify TMD stages [10]. JDD2021 is a widely adopted system in Japan and serves as a standardized framework for communication among healthcare professionals. Percentages of responses were calculated for single-choice questions, and frequency distributions were tabulated for multiple-choice items. For continuous variables, the median and interquartile range were reported. Descriptive statistics were presented in tables and figures, as appropriate. To assess differences in the distribution of energy content based on the presence or absence of multidisciplinary teams, the chi-squared test was applied. All statistical analyses were conducted using SPSS version 27.0 (IBM Corp., Armonk, NY), and a p-value of <0.05 was considered statistically significant.

JDD2021

JDD2021 is a classification proposed by the Japanese Society of Dysphagia Rehabilitation. JDD2021 is classified into five stages: code 0t (thickened), code 0j (jelly), code 1j (jelly that is more difficult than 0j), code 2-1 and 2-2 (purees classified according to grain properties), code 3 (solid foods that can be easily crushed with the tongue and palate), and code 4 (solid foods that can be easily crushed with the gums). The International Dysphagia Diet Standardization Initiative (IDDSI) is a framework used worldwide to classify TMDs [14]. A comparison between JDD2021 and IDDSI is presented in Appendix B and Appendix C.

## Results

A total of 5,376 hospitals were invited to participate in the survey, and 905 responded, resulting in a response rate of 16.8%. Among the hospitals responding, 857 (94.7%) provided TMDs, with general hospitals accounting for the largest proportion (728/857, 84.9%) by functional classification. In 511 out of 857 hospitals (59.6%), nutrition support for patients with dysphagia was provided by ward-assigned dietitians responsible for the care of those patients. In contrast, 229 hospitals (26.7%) reported having no clearly designated personnel in charge of such support. Additionally, only 57 (5.9%) of the registered dietitians held clinician certification from the Japanese Society of Dysphagia Rehabilitation, and only 16 (1.9%) were certified specialists of registered dietitians for dysphagia rehabilitation, indicating a limited presence of dietitians with specialized knowledge in this field. For nutritional assessment, more than 90% of hospitals reported using methods similar to those applied to patients without dysphagia (nutritional screening: 746/808, 92.3%; nutritional assessment: 791/841, 94.1%). Furthermore, 58.7% of hospitals reported not having a multidisciplinary team for feeding and swallowing management (Table 1).

**Table 1:** Characteristics of hospitals providing TMDs IQR, interquartile range; JSDR, Japanese Society of Dysphagia Rehabilitation; TMD, texture-modified diet *In charge of multiple wards and concurrently responsible for other duties such as food service management. †Each ward has a registered dietitian whose main task is nutrition support of the ward to which they are assigned. ‡Responses to this question were obtained from 808 facilities. §Responses were obtained from 841 facilities.

Variables	Overall
	n=857
Types of hospitals, n (%)	
General hospitals	728 (84.9)
Rehabilitation hospitals	44 (5.1)
Long-term hospitals	51 (6.0)
Psychiatric hospitals	11 (1.3)
Palliative care hospitals	1 (0.1)
Others	19 (2.2)
Unknown	3 (0.4)
Number of beds, median (IQR)	164 (95–304)
Length of hospital stay (day), median (IQR)	14 (11–20)
Registered dietitian, median (IQR)	
Total	4 (2–7)
Engaged in nutrition support	3 (2–5)
Nutrition support of patients with dysphagia, n (%)	
Registered dietitian in charge of hospital wards^*^	511 (59.6)
Registered dietitian assigned exclusively to hospital wards^†^	83 (9.7)
Not decided	229 (26.7)
Others	24 (2.8)
Unknown	10 (1.2)
Registered dietitian with JSDR-certified clinician, n (%)	57 (6.7)
Certified specialist of registered dietitian for dysphagia rehabilitation, n (%)	16 (1.9)
Nutrition screening methods for patients with dysphagia^‡^, n (%)	
Same method as other patients	746 (92.3)
Different method from other patients	37 (4.6)
Unknown	25 (3.1)
Nutritional assessment methods for patients with dysphagia^§^, n (%)	
Same method as other patients	791 (94.1)
Different method from other patients	23 (2.7)
Unknown	27 (3.2)
The presence or absence of a multidisciplinary team for dysphagia, n (%)	
Presence	336 (39.2)
Absence	503 (58.7)
Unknown	18 (2.1)

**Figure 1:**
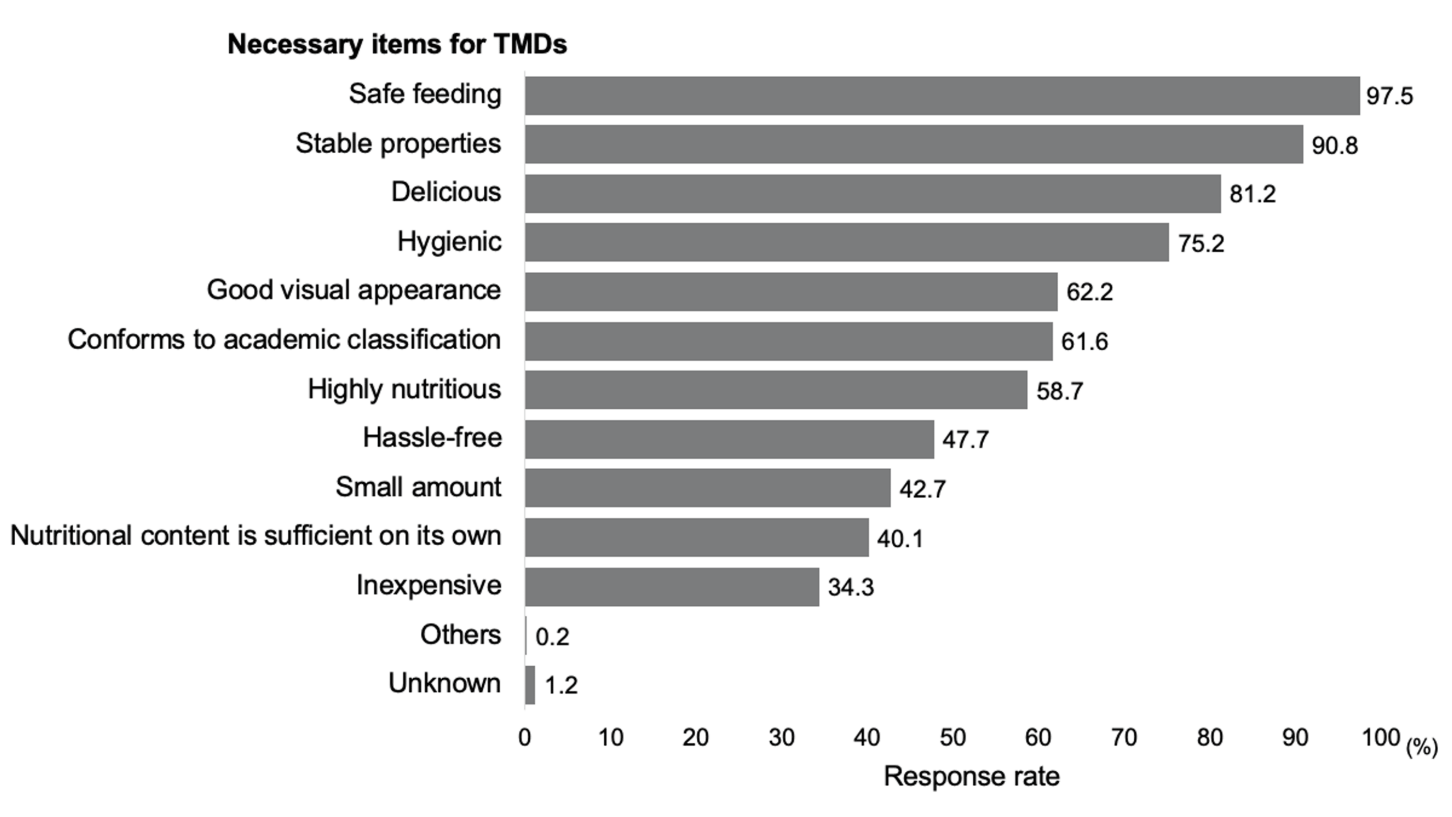
Necessary items for TMD as considered by dietitians (n=857, multiple answers allowed) TMD, texture-modified diet

Regarding the conditions required for TMDs, the safety of feeding (836/857, 97.5%) and stability of properties (778/857, 90.8%) were prioritized over aspects of nutritional value (highly nutritious: 503/857, 58.7%; small amount: 366/857, 42.7%; nutritional requirements are met: 344/857, 40.1%) (Figure 1).

**Figure 2:**
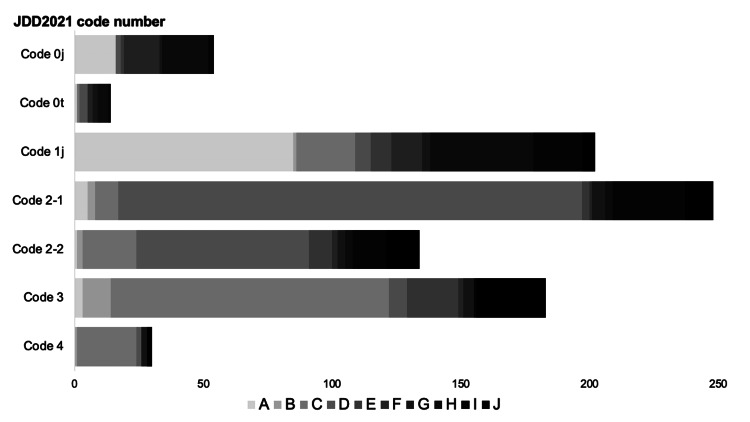
Mapping of the top 10 most frequently used meal names according to texture classification The meal names used in the hospitals are not consistent with the texture-modified diet levels classified by JDD2021. In this figure, the top 10 most frequently used meal names and their texture classifications are mapped. Because meal names are provided in Japanese, they were replaced with 10 letters from A to J to make them easier to understand. The name A was used for several levels of the adjusted swallowing diet (codes 0j, 0t, 1j, 2-1, 2-2, and 3). A similar occurrence is observed for other meal names and texture classifications. JDD2021, Japanese Dysphagia Diet 2021

In Japan, the JDD2021 code is used to classify the stage of TMDs, and the names of the TMDs often differ from one hospital to another. A survey revealed 60, 65, and 63 types of hospital-specific TMD names corresponding to codes 1j, 2-1, and 3, respectively. The most frequently used TMD names (D and G) span codes 0j-4 (Figure 2). However, only 319/857 (37.2%) hospitals shared dietary forms with other hospitals and facilities, and 727/857 (84.8%) encountered significant differences between TMD names and dietary forms. Nutritional information forms were prepared in 511/857 (59.6%) hospitals, with 459/511 (89.8%) incorporating the JDD2021 classification.

**Figure 3:**
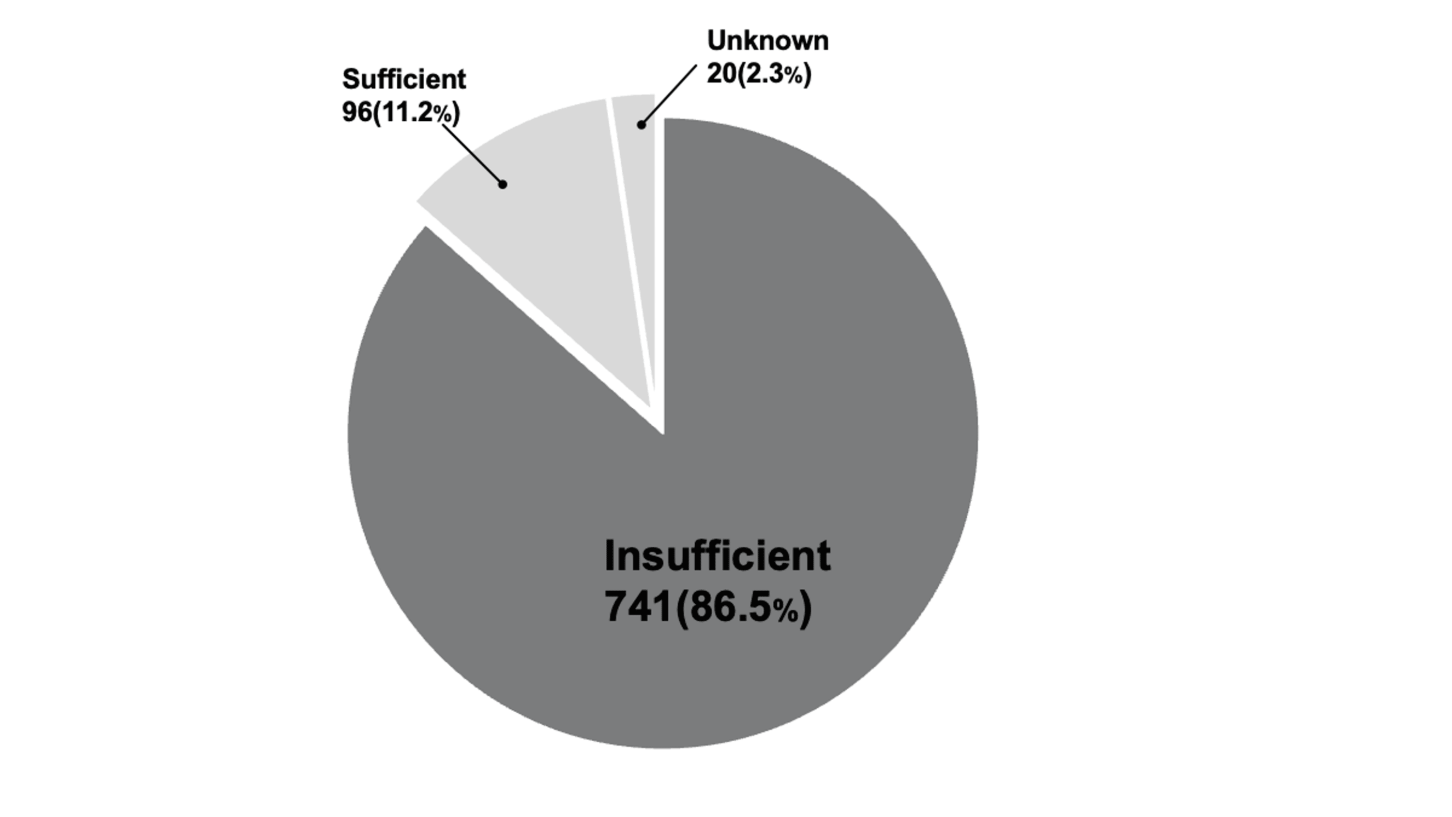
Dietitians' perception of the nutritional value of TMD TMD, texture-modified diet

**Figure 4:**
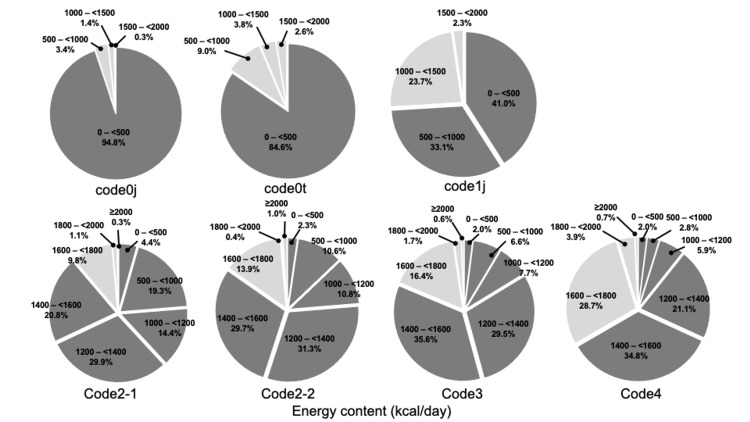
Distribution of actual energy content per day for TMDs TMD, texture-modified diet

**Figure 5:**
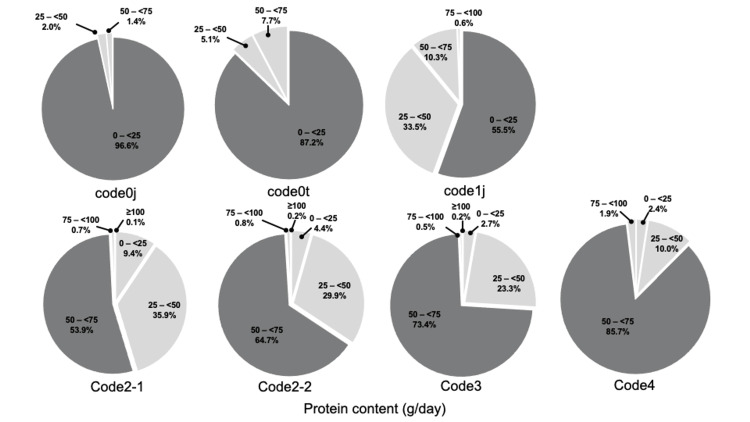
Distribution of actual protein content per day for TMDs TMD, texture-modified diet

**Figure 6:**
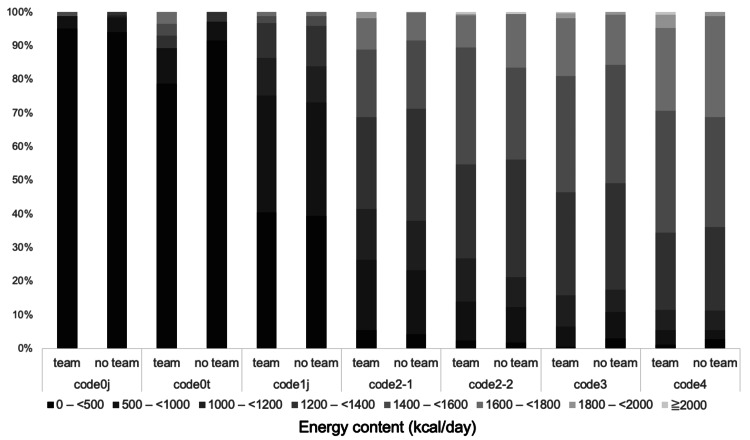
Comparison of actual energy distribution between hospitals with and without multidisciplinary teams

Approximately 90% (741/857, 86.5%) of the responding hospitals perceived the nutrient content of TMDs as inadequate (Figure 3). The actual energy content of the TMDs was insufficient, with the median ranging from 1,200 to 1,400 kcal/day for codes 2-4 (Table 2). The protein content increased progressively from 20.0 to 60.0 g/day for codes 1j-4 (Table 3). As a percentage, 74.1% of hospitals provided less than 1,000 kcal/day in code 1j, while over half provided less than 1,600 kcal/day in codes 2-1 to 4. More than half of the hospitals provided protein contents ranging from 50 to 75 g/day in codes 2-1 to 4. (Figures 4, 5).No statistically significant differences were observed in the distribution of energy content between hospitals with and without multidisciplinary teams (Figure 6).

**Table 2:** Energy content of TMDs (n=857, multiple answers allowed) IQR, interquartile range; JDD2021, Japanese Dysphagia Diet 2021; TMD, texture-modified diet

JDD2021 (meal)		Number of responses	Median (IQR)
Code	Title	n (%)	kcal/day
0j	Dysphagia rehabilitation food 0j	349 (40.7)	100 (50–200)
0t	Dysphagia rehabilitation food 0t	78 (9.1)	115 (30–300)
1j	Dysphagia diet 1j	620 (72.3)	600 (300–1000)
2-1	Dysphagia diet 2-1	755 (88.0)	1200 (1000–1400)
2-2	Dysphagia diet 2-2	518 (60.4)	1300 (1200–1500)
3	Dysphagia diet 3	665 (77.6)	1400 (1200–1500)
4	Dysphagia diet 4	540 (63.0)	1400 (1300–1600)

**Table 3:** Protein content of TMDs (n=857, multiple answers allowed) IQR, interquartile range; JDD2021, Japanese Dysphagia Diet 2021; TMD, texture-modified diet

JDD2021 (meal)		Number of responses	Median (IQR)
Code	Title	n (%)	g/day
0j	Dysphagia rehabilitation food 0j	349 (40.7)	0 (0–0)
0t	Dysphagia rehabilitation food 0t	78 (9.1)	0 (0–6.5)
1j	Dysphagia diet 1j	620 (72.3)	20 (10–35)
2-1	Dysphagia diet 2-1	755 (88.1)	50 (40–57)
2-2	Dysphagia diet 2-2	518 (60.4)	50 (45–60)
3	Dysphagia diet 3	665 (77.6)	55 (47–60)
4	Dysphagia diet 4	540 (63.0)	60 (50–62)

## Discussion

This nationwide questionnaire survey examined the nutrition support of patients with dysphagia in Japanese hospitals. Responses from across Japan were collected, and 857 hospitals providing TMDs were included in the analysis. The survey revealed three main findings. First, the nutrient content of TMDs is inadequate, as recognized by the nutrition departments. Second, there was a mismatch in the names and properties of TMDs among the hospitals. Third, few hospitals have a team approach to the nutrition support of dysphagia, and dietitians often work independently.

The nutrient content of TMDs is inadequate, and nutrition departments are aware of this issue. In this study, the median energy content of TMDs (1,200-1,400 kcal/day) was lower than the estimated requirements for older adults, particularly for men (1,800 kcal/day), suggesting a risk of undernutrition. Previous reports have shown inconsistent findings on whether TMDs meet energy and protein needs [7]. In many hospitals, limited time and resources hinder adequate nutritional evaluation of TMDs [15,16], and menu planning often prioritizes cost and staffing over nutrient content [15]. Our survey also revealed that safety and texture stability are prioritized over nutritional value. This aligns with the views of speech-language pathologists, who emphasize consistency and safety in TMDs [17]. As a result, TMDs may be regarded more as tools for swallowing management than as a means of nutritional support. However, given that patients on TMDs are at risk of reduced appetite and muscle mass [4], improving their nutritional value remains essential. Strategies such as food fortification or reshaping to mimic regular foods have shown promise [7], but practical constraints often limit implementation. As TMDs are a therapeutic diet for dysphagia management [18], their nutrient content should be reviewed to better support patients’ nutritional status.

This study revealed inconsistencies in the naming and classification of TMDs across hospitals. These discrepancies may pose challenges, particularly in clinical settings where continuity of care across institutions is required. Misalignment in TMD terminology and characteristics can lead to confusion among healthcare providers and patients, potentially compromising both nutrition support and swallowing safety [19,20]. Several factors may underlie these inconsistencies. Although JDD2021 offers a standardized framework [10], our findings showed that many hospitals used their own naming conventions or texture classifications, and that even when identical terms were used, the corresponding JDD2021 stages varied across institutions. One likely reason for this variation is the limited number of dietitians with formal qualifications in feeding and swallowing, as identified in our study. Previous research has shown that informal or insufficient training in this field may lead to misconceptions about texture modifications, potentially resulting in inappropriate preparation [21]. These factors may hinder the internal standardization of TMD practices and effective collaboration across hospitals. Therefore, the absence of clinical leaders with expertise in TMDs may be a key factor contributing to inconsistencies in terminology and classification.

Nutrition support for patients with dysphagia was primarily provided by dietitians assigned to hospital wards, with only a limited number of hospitals having established multidisciplinary teams. In addition, there was a notable shortage of dietitians with specialized qualifications in feeding and swallowing, indicating that expert-driven support systems remain underdeveloped. In this study, no statistically significant differences were observed in the distribution of nutritional values of TMDs between hospitals with and without multidisciplinary teams. However, previous studies have shown that multidisciplinary interventions can improve swallowing function and energy intake [22,23]. Furthermore, interventions by nutrition support teams (NSTs) have been reported to enhance oral and swallowing function in hospitalized older adults [22]. These findings suggest that leveraging existing team-based structures, such as NSTs, may offer a practical and effective approach to providing nutrition support for patients with dysphagia. Despite such evidence, the limited establishment of specialized multidisciplinary teams may be attributed to various organizational challenges, including insufficient staffing [16,18], limited resources [24,25], and a shortage of professionals with specialized expertise. Although this study did not assess actual nutrient intake and therefore cannot draw causal conclusions, it is plausible that multidisciplinary interventions could support the appropriate selection and application of TMDs tailored to individual patient conditions, which in turn may help increase nutrient intake. Moving forward, it will be important to investigate the development and effectiveness of more specialized team structures for the management of dysphagia.

The strength of this study is that responses were obtained evenly from hospitals throughout Japan, revealing, for the first time, the reality of the nutrition support of patients with dysphagia in Japanese hospitals. The results of this study will provide a basis for the further development of nutrition support in patients with dysphagia in Japan. This study had several limitations. First, the survey was requested from the nutrition department and did not ask about the respondents' job titles, years of experience, or areas of expertise. Therefore, the accuracy of the responses may vary. Second, although survey questionnaires were mailed to 5,376 hospitals nationwide, the response rate was 16.8%, and participation was based on voluntary consent. Hospitals that understood the purpose of the survey or were more motivated to improve nutrition support may have been more likely to respond. This may have introduced self-selection bias, potentially affecting the results and limiting the representativeness of the sample. Furthermore, since no stratified or random sampling was conducted, the generalizability of the findings is limited. However, the participating hospitals were distributed across various regions of Japan, which may partially mitigate this limitation. Third, to enhance the clarity and relevance of the questionnaire, we conducted multiple discussions and thoroughly reviewed and revised its content. However, formal reliability testing, such as test-retest reliability or internal consistency analysis, was not performed. Therefore, the reliability of the questionnaire responses was not statistically verified. Fourth, the study relied on self-reported data from hospital dietitians, including estimates of the nutrient content of TMDs. Such self-reported information may be subject to reporting bias, interpretation differences, or inaccuracies, particularly in hospitals without standardized nutrient evaluation procedures.

## Conclusions

This study revealed that the nutritional content of TMDs is often inadequate and that this issue is recognized by hospital nutrition departments. However, despite this awareness, substantial improvements have not been widely implemented in clinical practice. Inconsistencies in the naming and classification of TMDs across hospitals may further hinder effective communication and continuity of care. In addition, most hospitals do not have multidisciplinary teams dedicated to dysphagia care, and dietitians often manage patients independently. Although this study did not directly assess nutrient intake, previous research suggests that team-based approaches can improve nutritional outcomes. Addressing gaps in terminology, organizational structure, and practical implementation may contribute to enhancing nutrition support for patients with dysphagia.

## Figures and Tables

**Figure 7:**
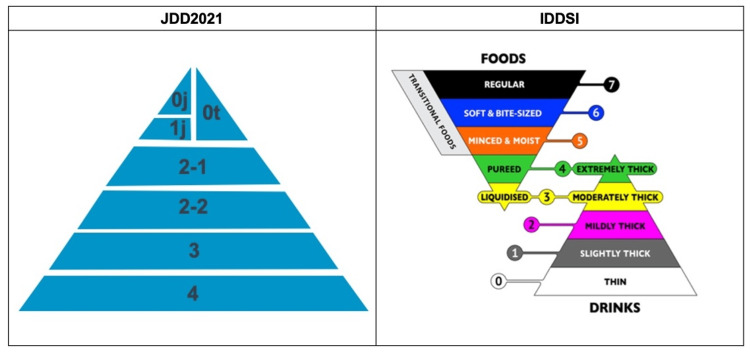
Framework of the Japanese Dysphagia Diet 2021 (JDD2021) and the International Dysphagia Diet Standardization Initiative (IDDSI) The source of the JDD2021 framework is as follows: Dysphagia Diet Committee of the Japanese Society of Dysphagia Rehabilitation; Kayashita J, Fujishima I, Fujitani J, et al.: The Japanese Dysphagia Diet of 2021 by the Japanese Society of Dysphagia Rehabilitation. Jpn J Compr Rehabil Sci. 2022, 13:64–77. 10.11336/jjcrs.13.64. The IDDSI framework is publicly available under the Creative Commons Attribution ShareAlike 4.0 International License. © The International Dysphagia Diet Standardisation Initiative 2019 (https://iddsi.org/standards/framework). Licensed under: https://creativecommons.org/licenses/by-sa/4.0/legalcode. Derivative works beyond language translation are not permitted. Accessed on April 3, 2025.

## Appendices

Appendix A 

**Table 4:** Comparison of the Japanese Dysphagia Diet 2021 (JDD2021) and the International Dysphagia Diet Standardization Initiative (IDDSI)

		JDD2021	IDDSI
Subject		Cases of dysphagia due to mid-career disorders in adults	Dysphagia of all ages, care settings, and cultures
Number of steps	Foods	5 steps	
	Liquid	3 steps	5 steps
How to describe the step		Indicated by code number. The code number consists of code 0, code 1, code 2, code 3, and code 4. In code 0 and 1, j and t were set as subclassifications, where j represents jelly and t represents thickening. In code 2, smooth and homogeneous foods are classified as 2‒1 and inhomogeneous foods containing soft grains as 2‒2.	Each step is classified by numbers, text labels, and color codes.
Texture		Solid foods that can be easily crushed with the gums (code 4)	Regular/easy to chew
		Formed solid foods that can be easily crushed with the tongue and palate (code 3)	Soft and bite-sized
		Pureed (code 2-1 and 2-2)	Pureed
		Jelly (code 0j: homogeneous jelly with minimal water separation, code 1j: jelly, pudding, and mousse-like foods)	Minced and moist
		Thickened liquid (code 0t)	Liquidized
Method of classification of diet form		Explanatory text and a quick reference table for each meal form	IDDSI Flow test, Fork Drip test, Fork Pressure test, Spoon Tilt test, Chopstick test, Finger test, food characteristics and other examples with photos
Liquid		Extremely thick	Extremely thick
		Moderately thick	Moderately thick
		Mildly thick	Mildly thick
			Slightly thick
			Thin
Method of classification of liquid		Line spread test, the residual amount by the syringe method	IDDSI Flow test, Fork Drip test, Spoon Tilt test, Fork Pressure test

Appendix B 

**Table 5:** Question content

Q1	Please specify the type of hospital function your hospital provides.
Q2	Please provide information about your hospital's diagnosis procedure combination (DPC) system.
Q3	If your hospital is a DPC hospital, please specify which wards are covered by the DPC system.
Q4	How many beds are there in your hospital?
Q5	Please provide the average length of stay in your hospital's acute phase general wards for the fiscal year 2022.
Q6	Please indicate whether your hospital employs a food service contracting company.
Q7	Please specify the scope of work outsourced to food service contractors at your hospital. (Multiple answers allowed)
Q8	Please specify the number of registered dietitians employed at your hospital.
Q9	Please specify the number of staff at your institution who meet each of the following criteria: 1. Registered dietitians who hold clinician certification from the Japanese Society of Dysphagia Rehabilitation (JSDR) 2. Certified specialists of registered dietitian for dysphagia rehabilitation
Q10	Please indicate if your hospital offers an adjusted texture-modified diet.
Q11	Please let us know if there are any barriers to introducing improved texture food preparation standards at the hospital.
Q12	Please indicate which of the following were barriers. (Multiple answers allowed)
Q13	Please indicate the duties of the registered dietitian for patients with dysphagia in your hospital. (Multiple answers are acceptable)
Q14	Please indicate whether you have a multidisciplinary team for dysphagia management.
Q15	Please tell us if you distinguish between the undernutrition screening method for patients with dysphagia and the method for all other patients.
Q16	Please tell us which positions perform undernutrition screening upon admission for patients with dysphagia.
Q17	Please tell us if you distinguish between the nutrition assessment method for patients with dysphagia and the method for all other patients.
Q18	Please tell us what types of positions perform nutrition assessment on admission for patients with dysphagia.
Q19	Please indicate the nutritional value of the texture-modified diets provided in your hospital. If your hospital offers several types of texture-modified diets and you think any of them are inadequate, please select "texture-modified diets alone are inadequate."
Q20	Please tell us about the conditions that you think are necessary for texture-modified diets, as applicable. (Multiple answers are acceptable)
Q21	Please indicate whether information regarding the nutrition support of patients with dysphagia is provided to transferring hospitals and other institutions.
Q22	Please tell us about the inclusion of the Japanese Dysphagia Diet 2013 or 2021 codes on the nutritional information form.
Q23	Does your hospital share its texture-modified diet standards with staff at other facilities involved in community and inter-facility collaboration?
Q24	Please indicate how you share your hospital's texture-modified diets standards with staff at other facilities involved in regional and inter-facility cooperation. (Multiple answers allowed)
Q25	Have you experienced a difference in the name or physical properties of the texture-modified diets between your hospital and other facilities?

Appendix C
